# Achieving Diabetes Remission Through Dietary Intervention: A 12‐Month Randomized Controlled Trial of Caloric‐Carbohydrate Restriction in Overweight Patients With Early‐Stage Type 2 Diabetes Mellitus

**DOI:** 10.1155/jdr/7230214

**Published:** 2026-05-28

**Authors:** Hongquan Wang, Qiong Wang, Jieqiong Chen, Yu Chen, Yanyao Tang

**Affiliations:** ^1^ Department of Intensive Care Medicine, Ningbo No.2 Hospital, Ningbo, Zhejiang, China; ^2^ Department of Endocrinology, Ningbo No.2 Hospital, Ningbo, Zhejiang, China

**Keywords:** calorie restriction, carbohydrate restriction, metabolic control, Type 2 diabetes remission, weight management

## Abstract

**Objective:**

We are aimed at evaluating the effects of a calorie‐restricted low‐carbohydrate diet (CR‐LCD) on diabetes remission, weight control, and metabolic parameters in overweight patients with early‐stage Type 2 diabetes mellitus (T2DM).

**Research Design and Methods:**

This 6‐month randomized intervention trial with a 12‐month follow‐up randomly assigned 68 adults with early‐stage T2DM to receive either a CR‐LCD or a control diet. Of the original cohort, data from 66 participants (two dropouts in the CR‐LCD group) were analyzed. The primary outcome was diabetes remission, defined as achieving a glycated hemoglobin (HbA1c) level < 6.5*%* without glucose‐lowering medications; secondary outcomes included anthropometric, glycemic, and lipid parameters.

**Results:**

The CR‐LCD group had a significantly higher 6‐month diabetes remission rate than the control group (62.50% vs. 35.29%, *χ* ^2^ = 4.885, *p* = 0.027). After multivariate adjustment, the intervention was associated with higher remission rates (odds ratio [OR] = 4.592, 95% confidence interval [CI]: 1.276–16.524; *p* = 0.020). At 12 months, between‐group comparisons following false discovery rate (FDR) correction revealed significant differences in body mass index (FDR − adjusted *p* = 0.006, *η* ^2^ *p* = 0.150), waist circumference (FDR − adjusted *p* = 0.040, *η* ^2^ *p* = 0.085), fasting blood glucose (FDR − adjusted *p* < 0.001, *r* = 0.613), 2‐h postprandial blood glucose (FDR − adjusted *p* = 0.040, *η* ^2^ *p* = 0.088), and high‐density lipoprotein cholesterol (FDR − adjusted *p* = 0.040, *η* ^2^ *p* = 0.095). No severe adverse events were reported.

**Conclusions:**

A CR‐LCD was effective in inducing diabetes remission in early‐stage T2DM, suggesting that it may offer a viable nonpharmacological management strategy for this condition.

**Trial Registration:**

Chinese Clinical Trial Registry: ChiCTR2600118189.

## 1. Introduction

Type 2 diabetes mellitus (T2DM) is a chronic metabolic disorder characterized by partially reversible *β*‐cell dysfunction and insulin resistance in its early stages [1]. Glycemic toxicity and lipotoxicity are key drivers of disease progression [2, 3]. Landmark trials, such as the DiRECT trial [4], have confirmed that intensive lifestyle interventions can facilitate diabetes remission, highlighting the substantial potential of non‐pharmacological strategies in the management of early‐stage T2DM [5].

Among nonpharmacological interventions, dietary modification plays a pivotal role in T2DM remission, and caloric restriction (CR) [6] and carbohydrate restriction (CCR) [7] are well‐established approaches. CR creates a negative energy balance that reduces adiposity and hepatic lipid accumulation, thereby alleviating lipotoxicity and improving insulin sensitivity [8]. Notably, even mild CR can substantially lower insulin requirements in patients with severe insulin resistance [9]. CCR reduces postprandial glycemic load and insulin demand while promoting fat oxidation, thereby directly mitigating glucotoxicity and supporting *β*‐cell rest [7, 10]. However, single dietary strategies have inherent limitations: CR is hindered by poor long‐term adherence owing to persistent hunger [11, 12], and CCR alone fails to address excess energy or sustain metabolic stability [13]. The combined calorie‐restricted low‐carbohydrate diet (CR‐LCD) integrates the complementary advantages of CR and CCR, offering unique synergistic benefits [14, 15]. Clinical evidence suggests that CR‐LCD, by simultaneously targeting excess energy and glycemic overload, may achieve more comprehensive metabolic regulation than single dietary approaches [16].

In this study, we are aimed at comparing the efficacy of a combined calorie‐ and carbohydrate‐restricted diet with that of a conventional diabetes diet in achieving diabetes remission, weight management, glycemic control, and lipid metabolism in overweight patients with early‐stage T2DM. We hypothesized that the combined intervention would yield higher remission rates and superior improvements in body weight, glycemic control, and lipid profiles. Targeting the early potentially reversible stages of T2DM may provide safe and effective metabolic benefits in clinical practice.

## 2. Methods

### 2.1. Study Design, Setting, and Oversight

This 12‐month randomized controlled trial (RCT) was conducted at the Standardized Metabolic Disease Management Center within the Department of Endocrinology of Ningbo No. 2 Hospital between June 2023 and June 2025. The Hospital Review Board approved the study protocol to ensure ethical compliance and transparency. Written informed consent was obtained from all participants before enrollment. The trial site, a regional diabetes care center, was selected for its robust patient‐recruitment capacity and its integrated MMC digital platform, which facilitated standardized data collection and follow‐up. The interventions were delivered by a multidisciplinary team, including an experienced principal investigator, an endocrinologist, a certified diabetes specialist nurse, and a registered dietitian specializing in diabetes nutrition. All team members possessed specific expertise in diabetes care and underwent protocol‐specific training to ensure consistent delivery of the interventions. The dietary guidance manuals used in this study were collaboratively developed by the team.

### 2.2. Participant Recruitment and Screening

Participants were recruited from our hospital′s endocrinology department and underwent a comprehensive screening process. Eligible individuals were adults diagnosed with T2DM within the past 5 years, with a body mass index (BMI) ≥ 24 kg/m^2^, and no prior history of severe diabetes‐related complications. Exclusion criteria comprised pregnancy, lactation, history of substance (alcohol/drug) abuse, dementia, acute infection, ≥ 5% weight loss in the preceding month, current use of weight‐loss medications, and insulin use within the past month. Participants were withdrawn if they failed to adhere to the standardized dietary protocol or if trial termination was deemed necessary based on clinical judgment.

**Figure 1:**
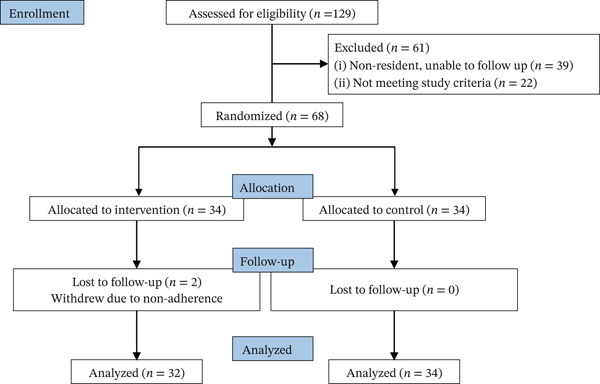
CONSORT diagram.

The sample size was estimated based on the primary outcome of diabetes remission rate, guided by two landmark RCTs, the DiRECT trial [4] and DIADEM‐I trial [5]. Both trials targeted overweight/obese individuals with early‐stage T2DM and employed structured dietary interventions, aligning well with the population characteristics of the present study. The reported remission rates were 46% (intervention) and 4% (control) in the DiRECT trial, and 61% (intervention) and 12% (control) in the DIADEM‐I trial. Given the uncertainty surrounding the effect size of the CR‐LCD used in this study, we adopted a conservative effect estimate. Specifically, the expected remission rate for the intervention group was set at the lowest of the two reference values, that is, 46%, and the expected remission rate for the control group was set at 12%, as this value most closely reflected the conventional dietary guidance used in the present study. The resulting absolute between‐group difference was 34 percentage points. To test this difference with 80% power at a two‐sided alpha level of 0.05 and accounting for an estimated 20% dropout rate, a minimum of 32 participants per group was required. Ultimately, 68 participants were included in this study. Two participants in the intervention group withdrew from the study because of consecutive failures to promptly submit their dietary records, body weight data, and blood glucose measurements (Figure 1).

### 2.3. Randomization and Blinding

This was a parallel‐group RCT with a 1:1 allocation ratio, designed as a superiority trial to test the hypothesis that a CR‐LCD is superior to a conventional energy‐balanced diet for achieving diabetes remission. Computer‐generated random sequences were used to ensure balanced participant allocation between the two arms. A research assistant with no role in eligibility screening, dietary advice, or outcome evaluation independently generated the randomization scheme. Control participants were allocated random Numbers 0–4, whereas participants in the intervention arm were assigned Numbers 5–9. Allocation specifics were sealed in opaque envelopes by a study coordinator and withheld from recruiters and data collectors. The group designation was not disclosed until a clerical staff member finalized the participant documentation. Research assistants, investigators, and statisticians were responsible for participant recruitment, data collection, and analysis.

Given the nature of dietary interventions, blinding of participants and intervention providers was not feasible. Consequently, outcome assessors were not blinded to the group assignment. To mitigate the potential for bias arising from this lack of blinding, the following procedures were implemented: (1) use of objective outcomes: The primary outcome and all key secondary outcomes were based on objective laboratory and anthropometric measurements, which are inherently less susceptible to assessment bias; (2) blinded data analysis: The data analyst remained blinded to group allocation until the completion of the primary analysis; and (3) separation of research roles: Participant recruitment, intervention delivery, outcome assessment, and data analysis were performed by independent personnel to prevent the cross‐contamination of roles.

### 2.4. Intervention

For participants randomized to the CR‐LCD intervention group, daily total caloric intake was individualized to match their basal metabolic rate (BMR) as measured by body composition analysis. Concurrently, the energy contribution of carbohydrates was restricted to 25%–30% of total energy intake, with protein accounting for 25%–35% and fat comprising 35%–40%. The study team provided structured guidance to support participants in implementing the dietary plan and using the *Calorie and Carbohydrate Restriction Dietary Guidance Manual* (Data S1). Guidance was delivered through two in‐person sessions and four virtual sessions, covering three core components: (1) introducing the diet plan′s rationale and macronutrient targets; (2) training on using the guidebook to design daily meal combinations aligned with energy and nutrient goals; and (3) providing practical adherence support, such as adapting dietary preferences to reduce restriction‐related burdens and managing hunger sensations. The intervention was maintained for 6 months. Throughout this period, participants were asked to record their daily dietary intake in the guidebook for at least 3 days per week. Team members offered ongoing check‐ins to ensure weekly adherence to the prescribed diet and address barriers (e.g., social events and cravings), minimizing attrition due to noncompliance.

The control group consumed an energy‐balanced diet. Target energy intake was calculated by integrating participants′ BMRs with energy expenditure from daily activities, with macronutrient contributions balanced as follows: 45%–60% from carbohydrates, 15%–20% from protein, and 20%–35% from lipids [17]. The research staff provided structured guidance to the participants for implementing this dietary plan, delivered through two face‐to‐face sessions and four virtual sessions.

To sustain weight loss achieved through the dietary intervention and ensure participant safety, participants were instructed to self‐monitor their weight weekly throughout this phase. Dietitians were instructed to intervene if participants met any of the following criteria: (1) weekly weight gain ≥ 2 kg, (2) weight loss exceeding 5% in a single month, (3) BMI < 18.5 kg/m^2^, or (4) serum albumin < 40 g/L. Concurrently, participants were instructed to perform self‐monitoring of blood glucose at least twice a week. Participants were instructed to contact an endocrinologist for assessment and management if any of the following abnormal glycemic conditions occurred: (1) fasting blood glucose (FBG) ≥ 7 mmol/L, (2) 2 − h postprandial blood glucose (2 h PBG) ≥ 10.0 mmol/L, or (3) random blood glucose ≤ 3.9 mmol/L or the presence of hypoglycemic symptoms. All antidiabetic medications were discontinued on the first day of the dietary intervention, consistent with protocols of landmark diabetes remission trials that excluded insulin users [4, 5]. If medication reintroduction was necessary, it was managed by endocrinologists in accordance with the *National Metabolic Management Center (MMC) Metabolic Disease Management Guidelines (2nd Edition)* [18].

Participants were withdrawn from the trial if they met any of the following nonadherence criteria: (1) fewer than three dietary records per week for 2 consecutive weeks, (2) failure to upload body weight data for 2 consecutive weeks, or (3) discontinuation of blood glucose monitoring for 1 week.

### 2.5. Outcomes

The primary outcome was diabetes remission, defined as a glycated hemoglobin (HbA1c) level of < 6.5% with no pharmacological therapy for T2DM for at least 3 months [19]. Secondary outcomes included weight loss, blood glucose control, and lipid management. Sociodemographic and disease‐related data were collected at baseline. Body weight, BMI, waist circumference, hip circumference, blood glucose, and lipid profiles were assessed longitudinally at baseline and at 6 and 12 months.

### 2.6. Statistical Analysis

Data were independently entered into IBM SPSS version 26.0 (IBM Corp., Armonk, New York, United States) by two researchers and cross‐checked for accuracy. Categorical variables were presented as frequencies (*n*) and percentages (%), and between‐group comparisons were performed using the chi‐square test. For continuous variables, normality was assessed using the Shapiro–Wilk test combined with visual inspection of histograms and Q–Q plots. Normally distributed variables were presented as mean ± standard deviation (SD). Between‐group comparisons at each time point were conducted using an independent‐samples *t*‐test, and within‐group changes from baseline to follow‐up were evaluated using paired‐sample *t*‐tests. Nonnormally distributed data were presented as medians (interquartile range [IQR], 25th–75th percentiles). Between‐group comparisons at each time point were performed using the Mann–Whitney *U* test, and within‐group changes from baseline to follow‐up were evaluated using the Wilcoxon signed‐rank test.

To control for the effect of baseline imbalance in waist circumference, the primary outcome (diabetes remission rate) was analyzed using logistic regression. Nested models were constructed, including unadjusted, adjusted for baseline waist circumference, and multivariable‐adjusted models, to evaluate the robustness of the intervention effect. Secondary outcomes were analyzed using analysis of covariance (ANCOVA). For variables that violated the normality assumption (hip circumference, fasting glucose, HbA1c, and triglycerides at postintervention time points), a nonparametric ANCOVA approach was employed. Dependent and covariate variables were rank‐transformed, a rank regression model was fitted to compute adjusted residuals, and the residuals were then compared between groups using the Mann–Whitney *U* test. The remaining secondary outcomes that met the assumptions of normality were analyzed using standard ANCOVA. To account for potential Type I error inflation due to multiple comparisons, the *p-*values for secondary outcomes (derived from ANCOVA or non‐parametric ANCOVA) were adjusted for the false discovery rate (FDR) using the Benjamini–Hochberg method. All statistical tests were two‐tailed, and statistical significance was defined as *p* < 0.05. The CONSORT 2025 checklist for this trial is provided in Data S2.

## 3. Results

### 3.1. Baseline Characteristics of Participants

A total of 68 participants with overweight and early‐stage T2DM were enrolled and randomly assigned to either the intervention group (CR‐LCD) or the control group (target energy‐balanced diet). Two participants in the intervention group dropped out owing to difficulty adhering to the dietary plan, resulting in 32 evaluable participants in the intervention group and 34 in the control group for the final analysis. This yielded a dropout rate of 5.88% (2/34) in the intervention group.

Baseline clinical, demographic, and metabolic characteristics of study participants are summarized in Table 1. The two groups were well balanced at baseline, except for waist circumference (*p* = 0.027). Participants in the control group had a significantly greater baseline waist circumference than those in the intervention group.

**Table 1:** Baseline characteristics of study participants.

Characteristic	Intervention (*n* = 32)	Control (*n* = 34)	*p* value
Age (years)	47.91 ± 9.84	46.35 ± 12.82	0.584
Sex	Male: 21 (65.62%)	Male: 24 (70.59%)	0.665
	Female: 11 (34.38%)	Female: 10 (29.41%)	
Duration of diabetes (months)	36.53 ± 18.40	39.41 ± 18.43	0.528
C‐P (nmol/L)	1.65 (1.25–2.42)	1.92 (1.14–2.85)	0.581
2 h C‐P (nmol/L)	6.38 ± 4.11	7.40 ± 4.31	0.328
Weight (kg)	75.05 ± 13.56	77.30 ± 11.42	0.468
BMI (kg/m^2^)	27.11 ± 2.58	28.26 ± 3.07	0.106
WC (cm)	91.45 ± 10.06	96.69 ± 8.70	0.027
HC (cm)	96.50 (93.25–103.83)	99.00 (93.35–102.88)	0.715
FBG (mmol/L)	5.84 (4.75–7.18)	5.47 (4.61–6.61)	0.449
2 h PBG (mmol/L)	11.54 ± 3.81	11.72 ± 3.49	0.841
HbA1c (%)	10.05 (7.83–11.35)	9.90 (7.98–12.30)	0.974
TC (mmol/L)	4.85 ± 1.31	4.79 ± 1.54	0.863
TG (mmol/L)	1.37 (0.90–2.00)	1.62 (0.95–2.18)	0.408
HDL‐C (mmol/L)	1.20 ± 0.26	1.08 ± 0.26	0.070
LDL‐C (mmol/L)	3.03 ± 1.05	2.93 ± 0.77	0.659
Antidiabetic medication use	15 (46.88%)	17 (50.00%)	0.800

*Note:* Data are presented as mean ± SD, median (IQR), or *n* (%), as appropriate.

Abbreviations: 2‐h C‐P, 2‐h C‐peptide; 2‐h PBG, 2‐h postprandial blood glucose; C‐P, C‐peptide; FBG, fasting blood glucose; HC, hip circumference; HDL‐C, high‐density lipoprotein cholesterol; LDL‐C, low‐density lipoprotein cholesterol; TC, total cholesterol; TG, triglycerides; WC, waist circumference.

### 3.2. Primary Outcome: T2DM Remission Rate

The unadjusted remission rates were 62.50% (20/32) and 35.29% (12/34) in the intervention and control groups, respectively, demonstrating a significant difference between groups (*χ* ^2^ = 4.885, *p* = 0.027). Nested logistic regression models were constructed to account for the baseline imbalance in waist circumference (Table 2). The intervention effect remained significant after adjusting for baseline waist circumference alone (Model B: odds ratio [OR] = 3.808, 95% confidence interval [CI]: 1.283 to 11.305, *p* = 0.016). Furthermore, the association persisted after additional adjustment for age, baseline HbA1c level, and diabetes duration (Model C: OR = 4.592, 95% CI: 1.276 to 16.524, *p* = 0.020).

**Table 2:** Logistic regression models for diabetes remission rate.

Variable	Model A	Model B	Model C
Intervention (vs. control)	3.056 (1.120–8.335) *p* = 0.029	3.808 (1.283–11.305) *p* = 0.016	4.592 (1.276–16.524) *p* = 0.020
Baseline WC (per cm)	—	1.037 (0.980–1.097) *p* = 0.280	1.025 (0.957–1.097) *p* = 0.482
Age (per year)	—	—	0.957 (0.905–1.012) *p* = 0.126
Baseline HbA1c (per %)	—	—	1.111 (0.871–1.418) *p* = 0.395
Diabetes duration (per month)	—	—	0.951 (0.918–0.986) *p* = 0.006
Model fit			
Sample size (*N*)	66	66	66
−2 Log likelihood	86.489	84.845	76.830
Hosmer–Lemeshow *p* value	—	0.876	0.402

*Note:* Model A was not adjusted. Model B was adjusted for baseline waist circumference (WC). Model C was adjusted for baseline WC, age, HbA1c levels, and duration of diabetes. Data are presented as odds ratios (95% confidence interval).

### 3.3. Secondary Outcomes: Comparison of Weight Control, Blood Glucose, and Lipid Indicators

After adjusting for baseline waist circumference imbalance, the results of the secondary outcome analyses at 6 and 12 months post‐intervention are presented in Table 3. At 6 months, participants in the CR‐LCD group exhibited significantly lower values than those in the control group for the following indicators: BMI (adjusted mean difference [AMD] = −1.378 kg/m^2^; 95% CI: −2.719 to −0.037, *p* = 0.044), waist circumference (AMD = −3.925 cm, 95% CI: −7.402 to −0.448, *p* = 0.028), FBG (*Z* = −2.771, *p* = 0.006), and 2‐h PBG (AMD = −1.848 mmol/L, 95% CI: −3.462 to −0.234, *p* = 0.026). After FDR correction for multiple comparisons, none of these between‐group differences remained significant at 6 months.

**Table 3:** Results of ANCOVA or nonparametric ANCOVA for secondary outcome variables at 6 and 12 months.

Variable	Time	Intervention (*n* = 32)	Control (*n* = 34)	Statistic	*p* value	Effect size	FDR
Weight (kg)	6 m	70.10 ± 14.32	75.26 ± 10.39	0.171 (−3.956 to 4.298)	0.934	*η* ^2^ *p* < 0.001	0.934
	12 m	69.21 ± 14.69	76.72 ± 11.51	−1.768 (−5.990 to 2.455)	0.406	*η* ^2^ *p* = 0.011	0.447
BMI (kg/m^2^)	6 m	25.24 ± 3.18	27.89 ± 3.71	−1.378 (−2.719 to −0.037)	0.044	*η* ^2^ *p* = 0.063	0.121
	12 m	24.87 ± 3.17	28.24 ± 3.55	−2.020 (−3.229 to −0.812)	0.001	*η* ^2^ *p* = 0.150	**0.006**
WC (cm)	6 m	88.38 ± 8.86	96.00 ± 9.97	−3.925 (−7.402 to −0.448)	0.028	*η* ^2^ *p* = 0.075	0.103
	12 m	88.20 ± 9.61	96.65 ± 10.75	−4.035 (−7.365 to −0.705)	0.018	*η* ^2^ *p* = 0.085	**0.040**
HC (cm)	6 m	94.60 (89.93–102.73)	97.25 (92.38–102.03)	0.532	0.594	*r* = 0.065	0.817
	12 m	93.50 (88.25–99.13)	98.35 (93.08–103.25)	−0.982	0.326	*r* = 0.121	0.398
TC (mmol/L)	6 m	4.17 ± 1.03	4.28 ± 1.14	‐0.088 (−0.646 to 0.469)	0.778	*η* ^2^ *p* = 0.001	0.923
	12 m	4.57 ± 1.11	4.30 ± 1.09	0.342 (−0.222 to 0.906)	0.230	*η* ^2^ *p* = 0.023	0.316
TG (mmol/L)	6 m	1.03 (0.66–1.70)	1.58 (1.10–2.78)	−1.796	0.072	*r* = 0.221	0.158
	12 m	1.08 (0.76–1.82)	1.69 (1.19–2.65)	−2.194	0.028	*r* = 0.270	0.051
HDL‐C (mmol/L)	6 m	1.32 ± 0.28	1.19 ± 0.36	0.123 (−0.043 to 0.290)	0.144	*η* ^2^ *p* = 0.034	0.264
	12 m	1.36 ± 0.30	1.17 ± 0.30	0.197 (0.043 to 0.350)	0.013	*η* ^2^ *p* = 0.095	**0.040**
LDL‐C (mmol/L)	6 m	2.32 ± 0.77	2.40 ± 0.75	−0.040 (−0.430 to 0.350)	0.839	*η* ^2^ *p* = 0.001	0.923
	12 m	2.50 ± 0.76	2.40 ± 0.71	0.116 (−0.261 to 0.493)	0.541	*η* ^2^ *p* = 0.006	0.541
HbA1c (%)	6 m	6.05 (5.63–6.60)	6.20 (5.65–6.83)	−1.078	0.281	*r* = 0.133	0.442
	12 m	6.30 (5.70–6.98)	6.35 (5.80–7.00)	−1.245	0.213	*r* = 0.153	0.316
FBG (mmol/L)	6 m	5.23 (4.74–5.91)	6.34 (5.38–7.15)	−2.771	0.006	*r* = 0.341	0.066
	12 m	5.15 (4.66–6.11)	7.02 (6.10–7.89)	−4.978	<0.001	*r* = 0.613	**< 0.001**
2 h PBG (mmol/L)	6 m	8.61 ± 3.20	10.28 ± 3.10	−1.848 (−3.462 to −0.234)	0.026	*η* ^2^ *p* = 0.077	0.103
	12 m	8.60 ± 2.58	10.18 ± 2.69	−1.672 (−3.029 to −0.315)	0.017	*η* ^2^ *p* = 0.088	**0.040**

*Note:* Data are presented as mean ± standard deviation or median (interquartile range, IQR). Statistics: adjusted mean difference (95% CI) (parametric) or *Z*‐statistic (nonparametric). Effect sizes are expressed as partial eta‐squared (*η* ^2^ *p*) for parametric tests and Pearson′s correlation coefficient (*r*) for nonparametric tests. The values presented in boldface serve exclusively to indicate statistical significance and do not carry any additional interpretive or substantive implications.

Abbreviations: 2‐h PBG, 2‐h postprandial blood glucose; FBG, fasting blood glucose; FDR, false discovery rate; HC, hip circumference; HDL‐C, high‐density lipoprotein cholesterol; LDL‐C, low‐density lipoprotein cholesterol; TC, total cholesterol; TG, triglycerides; WC, waist circumference.

At the 12‐month follow‐up, between‐group differences remained significant in the intervention group for BMI (AMD = −2.020 kg/m^2^, 95% CI: −3.229 to −0.812, *p* = 0.001, *η* ^2^ *p* = 0.150), waist circumference (AMD = −4.035 cm, 95% CI: −7.365 to −0.705, *p* = 0.018, *η* ^2^ *p* = 0.085), FBG (*Z* = −4.978, *p* < 0.001, *r* = 0.613), and 2‐h PBG (AMD = −1.672 mmol/L, 95% CI: −3.029 to −0.315, *p* = 0.017, *η* ^2^ *p* = 0.088). Significant between‐group differences were also observed in triglycerides (*Z* = −2.194, *p* = 0.028, *r* = 0.270) and high‐density lipoprotein cholesterol (HDL‐C) (AMD = 0.197 mmol/L, 95% CI: 0.043 to 0.350, *p* = 0.013, *η* ^2^ *p* = 0.095). After FDR correction for multiple comparisons, between‐group differences remained significant for BMI (FDR − adjusted *p* = 0.006), waist circumference (FDR − adjusted *p* = 0.040), FBG (FDR − adjusted *p* < 0.001), 2‐h PBG (FDR − adjusted *p* = 0.040), and HDL‐C (FDR − adjusted *p* = 0.040).

The intragroup comparisons of changes from baseline to 6 and 12 months are presented in Table 4. Compared with baseline values, the intervention group showed significant reductions in weight, BMI, waist circumference, and hip circumference at both 6 and 12 months (all *p* < 0.05). However, the control group showed no significant changes in these measurements at any time point.

**Table 4:** Intragroup differences in secondary outcome variables.

Variable	*p* value (baseline vs. 6 months)		*p* value (baseline vs. 12 months)	
	Intervention	Control	Intervention	Control
Weight (kg)	**< 0.001**	0.140	**< 0.001**	0.641
BMI (kg/m^2^)	**< 0.001**	0.540	**< 0.001**	0.972
WC (cm)	**0.004**	0.643	**0.001**	0.976
HC (cm)	**< 0.001** ^**a**^	0.491 ^a^	**< 0.001** ^**a**^	0.971^a^
FBG (mmol/L)	0.140 ^a^	**0.027** ^**a**^	**0.025** ^**a**^	**< 0.001** ^**a**^
PBG (mmol/L)	**0.001**	0.076	**< 0.001**	**0.049**
HbA1c (%)	**< 0.001** ^a^	**< 0.001** ^a^	**< 0.001** ^a^	**< 0.001** ^a^
TC (mmol/L)	**0.004**	**0.030**	0.177	**0.043**
TG (mmol/L)	0.395 ^a^	0.235 ^a^	0.322^a^	0.218^a^
HDL‐C (mmol/L)	**0.005**	**0.042**	**< 0.001**	**0.024**
LDL‐C (mmol/L)	**< 0.001**	**< 0.001**	**0.001**	**0.001**

*Note:* All others used paired‐sample *t*‐tests. The values presented in boldface serve exclusively to indicate statistical significance and do not carry any additional interpretive or substantive implications.

Abbreviations: FBG, fasting blood glucose; HC, hip circumference; HDL‐C, high‐density lipoprotein cholesterol; LDL‐C, low‐density lipoprotein cholesterol; PBG, postprandial blood glucose; TC, total cholesterol; TG, triglycerides; WC, waist circumference.

^a^Wilcoxon signed‐rank test.

Regarding glycemic control, both groups showed significant improvements in HbA1c levels at 6 and 12 months (all *p* < 0.001). The intervention group exhibited a significant reduction in FBG at 12 months (*p* = 0.025), although no change was observed at 6 months. Conversely, the control group showed significant increases in FBG at both time points (6 months: *p* = 0.027; 12 months: *p* < 0.001). For 2‐h PBG, the intervention group demonstrated significant reductions at 6 (*p* = 0.001) and 12 months (*p* < 0.001). In contrast, the control group showed a significant increase at 12 months (*p* = 0.049), with no change at 6 months.

Regarding lipid profile indicators, the intervention group showed a significant reduction in total cholesterol at 6 months (*p* = 0.004) but no change at 12 months. Conversely, the control group showed significant reductions in total cholesterol at both time points (6 months: *p* = 0.030; 12 months: *p* = 0.043). No significant changes in triglyceride levels were observed in either group. The intervention group exhibited significant increases in HDL‐C at 6 months (*p* = 0.005) and 12 months (*p* < 0.001), whereas the control group showed significant increases at both time points (6 months: *p* = 0.042; 12 months: *p* = 0.024). Additionally, both groups demonstrated significant reductions in low‐density lipoprotein cholesterol (LDL‐C) levels at 6 and 12 months (all *p* < 0.001).

No episodes of severe hypoglycemia (defined as symptomatic hypoglycemia with blood glucose ≤3.9 mmol/L) or diabetic ketoacidosis were observed in either group during the intervention period.

## 4. Discussion

In the present study, the diabetes remission rate at 6 months was markedly higher in the calorie‐ and carbohydrate‐restricted diet group than in the conventional diet group (62.50% vs. 35.29%). Notably, the control group had a significantly larger baseline waist circumference than the intervention group (96.69 vs. 91.45 cm, *p* = 0.027). Waist circumference is a well‐established clinical marker of abdominal obesity and visceral adiposity [20], and excess visceral fat is closely associated with insulin resistance, chronic low‐grade inflammation, and poorer metabolic outcomes in patients with T2DM [21, 22]. The larger baseline waist circumference of the control group suggests a more severe underlying metabolic disturbance, which theoretically reduces the likelihood of diabetes remission under conventional management. Baseline waist circumference was adjusted for in the primary outcome analysis using multivariable logistic regression. After adjustment, the intervention group maintained a notable advantage in diabetes remission, with an increase in the effect estimate relative to the unadjusted model. This indicates that the baseline waist circumference imbalance did not inflate the intervention effect; rather, it may have led to an underestimation of the intervention effect in the unadjusted analysis. Accordingly, if the two groups had comparable baseline waist circumferences, the advantages observed in the intervention group might have been even more pronounced.

The diabetes remission rate in the CR‐LCD group was comparable to the 61% rate reported in the DIADEM‐I trial [5]. Both studies precisely targeted overweight individuals with early‐stage T2DM, albeit with differing intervention intensities. The DIADEM‐I trial employed a total diet replacement (TDR) regimen characterized by strict energy restriction. This approach was associated with a dropout rate of approximately 21%, primarily during the TDR phase. In contrast, the present study recorded only two dropouts, yielding a dropout rate of 5.88%; no dropouts were attributed to dietary intolerance. These findings suggest that compared with TDR, the CR‐LCD approach may be associated with lower dropout rates and better adherence in real‐world settings. This difference may stem from the allowance of the CR‐LCD model for everyday food choices and its relatively moderate intervention intensity [23].

In recent years, the number of low‐carbohydrate dietary intervention studies targeting Asian populations has increased steadily [24–26]. A systematic review and meta‐analysis conducted by Hironaka et al. [27], which included six RCTs in East Asian populations, reported that a low‐carbohydrate diet was more effective than a control diet in reducing HbA1c and BMI, with low heterogeneity between studies. Of the six, five studies were completed over a 6‐month period, indicating that the short‐term efficacy of a low‐carbohydrate diet is well established. A study by Tian et al. supports this perspective [28]. However, the studies included in these systematic reviews did not consider diabetes remission as a primary endpoint. A more directly comparable study is a multicenter RCT conducted by Liu et al. [29], which enrolled a highly similar population of Chinese patients with early‐stage T2DM (duration < 6 years, BMI ≥ 24 kg/m^2^, no insulin use). This study reported a 12‐month remission rate of 44% with dapagliflozin combined with moderate calorie restriction. In the present study, a 62.5% remission rate was achieved through calorie and carbohydrate restriction without pharmacological intervention, accompanied by a similar magnitude of weight loss (approximately 5.8 kg vs. 5.0 kg in the study by Liu et al.). Subgroup analysis of the DiRECT trial showed that remission rates varied among participants with similar weight loss [4], suggesting that factors beyond the magnitude of weight reduction may contribute to diabetes remission [8]. Future studies may directly compare different dietary patterns or employ mediation analysis to explore the factors associated with higher remission rates under conditions of comparable weight loss.

In addition to the primary outcome of diabetes remission, this study quantified the metabolic benefits of the CR‐LCD intervention across three dimensions: anthropometric measurements, glycemic control, and lipid metabolism. Longitudinal analysis showed that, in the CR‐LCD group, body weight, BMI, waist circumference, and hip circumference were markedly reduced from baseline at both 6 and 12 months. In contrast, no significant changes were observed in the control group. Sun et al. demonstrated that combined calorie and carbohydrate restriction was more effective in reducing body weight and waist circumference than either calorie restriction or a low‐carbohydrate diet alone in non‐diabetic individuals with overweight/obesity [16]. The present study extends this approach to individuals with early‐stage T2DM and demonstrates that a CR‐LCD effectively reduces body weight, BMI, waist circumference, and hip circumference within 6 months.

In the intervention group, HbA1c, FBG, and postprandial blood glucose levels showed a downward trend at 6 months. However, the within‐group improvement in FBG levels did not reach significance until 12 months. This temporal pattern was not consistent with the findings of a systematic review by Goldenberg et al. [30], which reported significant improvements in both HbA1c and FBG levels at 6 months, with the benefits attenuating by 12 months. The discrepancies may be attributable to differences in intervention design, medication management, and baseline characteristics. Some studies included in the review by Goldenberg et al. allowed the continued or tapered use of glucose‐lowering medications. In contrast, all such medications were discontinued at baseline in the present study, making glycemic changes more directly reflective of dietary effects. Furthermore, the baseline HbA1c level in the intervention group in this study was 10.05%, which was higher than the baseline HbA1c levels (7.6%–8.3%) reported in the individual studies included in the review by Goldenberg et al., indicating more severe baseline dysglycemia in the present study population. This may partly explain why a significant improvement in FBG was observed only at 12 months, as achieving a substantial reduction from a higher baseline level may require a longer intervention period. Additionally, FBG levels in the control group showed a persistently increasing trend, rising from 5.47 mmol/L at baseline to 7.02 mmol/L at 12 months. This pattern aligns with the natural progression of early‐stage T2DM and indirectly supports the role of a CR‐LCD in preventing the deterioration of FBG levels.

After FDR correction, no significant differences in lipid metabolism were observed between the groups at 6 months. Nevertheless, the intervention group exhibited favorable trends, including reductions in total cholesterol and LDL‐C levels, and an increase in HDL‐C. Changes in HDL‐C and LDL‐C levels were sustained at 12 months, with HDL‐C levels in the intervention group significantly higher than those in the control group. Another study also reported favorable changes in lipid profiles, with the CR‐LCD group showing more pronounced reductions in triglyceride levels and maintenance of HDL‐C levels [15]. Although the specific lipid parameters that reached significance differed between the two studies, the overall direction and selectivity of the lipid improvements were consistent, providing evidence for the positive effect of the CR‐LCD on lipid profiles.

Although no significant between‐group differences in secondary outcomes were observed at 6 months, the CR‐LCD group exhibited consistently more favorable outcomes than the control group at 12 months for BMI, waist circumference, HDL‐C, FBG, and 2‐h PBG. This observation aligns with existing evidence indicating that metabolic improvements from lifestyle interventions may have a delayed onset or may require extended follow‐up to stabilize [31]. A key contributing factor to the delayed between‐group differences at 6 months may be the improvement in metabolic parameters often seen in control groups of lifestyle intervention trials after receiving standardized health guidance, consistent with findings from large randomized controlled trials such as the Look AHEAD trial [32]. Specifically, such improvements in the control group can dilute the short‐term between‐group effects of intensive interventions, which may explain why the beneficial effects of the CR‐LCD were not fully evident at the 6‐month time point in the present study. Furthermore, a meta‐analysis of 27 clinical trials found that the maximal effects of lifestyle interventions on body weight may peak several months after the intervention has ended [33]. The between‐group differences observed at 12 months in this study are consistent with this phenomenon, further supporting the notion that the efficacy of lifestyle interventions, including CR‐LCD, may become more pronounced and stable over time.

Although these findings support the clinical efficacy of the CR‐LCD in real‐world settings, the associated methodological trade‐offs warrant cautious interpretation. This open‐label trial included unblinded participants, intervention providers, and outcome assessors. In theory, unblinded assessors could introduce assessment bias, as knowledge of allocation may influence measurement procedures, particularly for anthropometric outcomes, where minor variations in technique could occur. We implemented two pre‐specified mitigation strategies: (1) all primary and key secondary outcomes were objectively measured (laboratory and anthropometric assessments), with meta‐epidemiological evidence indicating that unblinded assessment primarily exaggerates effects on subjective outcomes, with a substantially smaller impact on objective measures [34]; and (2) strict separation of research roles and blinded data analysis were employed to prevent bias and cross‐contamination. These measures likely limit residual bias in the primary outcome of diabetes remission, given that HbA1c is a machine‐measured laboratory endpoint, the effect estimate is large (adjusted OR = 4.592), and the observed effect aligns with established physiological mechanisms. Nevertheless, the complete elimination of bias cannot be guaranteed, particularly for secondary outcomes that require assessor judgment, which should be interpreted with caution. Future trials should incorporate independent blinded assessors or centralized laboratories to strengthen methodological rigor.

In addition to the issues related to blinding, some other limitations need to be acknowledged. First, we did not use a validated quantitative tool to assess dietary adherence. Although we implemented comprehensive supervision procedures, including mandatory weekly dietary records, continuous follow‐up by medical staff, and clear withdrawal criteria for nonadherence, we did not employ a formal quantitative adherence scale. Future dietary intervention trials should incorporate validated tools for a more precise and systematic evaluation of dietary compliance, thereby strengthening the interpretability of intervention effects. Second, this was a single‐center study conducted at a specialized metabolic management center in eastern China, and all participants were overweight patients with early‐stage T2DM. Consequently, the findings may not be directly generalizable to other ethnic groups, patients with longer disease duration, or patients with different metabolic phenotypes. Third, the relatively short 12‐month follow‐up period limits our ability to assess the long‐term sustainability of diabetes remission and its metabolic benefits. Finally, despite the notable clinical outcomes, the lack of mechanistic measurements (e.g., pancreatic and hepatic fat quantification and indirect calorimetry) precludes definitive conclusions regarding the underlying biological pathways responsible for these improvements.

## 5. Conclusions

This 12‐month RCT demonstrated that compared with a conventional energy‐balanced diet, a CR‐LCD may markedly increase the diabetes remission rate in overweight patients with early‐stage T2DM. Furthermore, at 12 months postintervention, the CR‐LCD group showed superior outcomes compared with the control group for BMI, waist circumference, fasting and postprandial blood glucose, and HDL‐C levels. These findings suggest that a dietary strategy combining carbohydrate and calorie restriction based on everyday food choices may represent a viable nonpharmacological treatment option for inducing diabetes remission in this patient population.

## Author Contributions

Yu Chen and Yanyao Tang contributed equally as co‐corresponding authors.

## Funding

This work was supported by the Medical Scientific Research Foundation of Zhejiang Province (Grant No. 2023KY1099).

## Conflicts of Interest

The authors declare no conflicts of interest.

## Supporting Information

Additional supporting information can be found online in the Supporting Information section.

## Supporting information


**Supporting Information 1** Data S1: Calorie and Carbohydrate Restriction Dietary Guidance Manual. This document functioned as the central protocol for the “caloric‐carbohydrate restricted dietary intervention” implemented in this study. It specifies standard portion sizes and caloric values for common food categories. It includes exemplar meal plans customized to varying daily energy needs, thus ensuring the standardization and reproducibility of the intervention protocol.

**Supporting Information 2** Data S2: CONSORT 2025 checklist. The CONSORT 2025 checklist has been completed in accordance with our study report. It clearly delineates the manuscript page numbers corresponding to each reporting item, demonstrating the study′s strict adherence to international reporting standards.

## Data Availability

The data supporting the findings of this study are available from the corresponding author upon request.
