# Supplementary material for: Achieving Diabetes Remission Through Dietary Intervention: A 12‐Month Randomized Controlled Trial of Caloric‐Carbohydrate Restriction in Overweight Patients With Early‐Stage Type 2 Diabetes Mellitus
Source: J Diabetes Res. 2026 May 28;2026:7230214. doi: 10.1155/jdr/7230214 (PMC13240505; doi:10.1155/jdr/7230214)
Supplement: Supplementary file 1 — Supporting Information 1 Data S1: Calorie and Carbohydrate Restriction Dietary Guidance Manual. This document functioned as the central protocol for the “caloric‐carbohydrate restricted dietary intervention” implemented in this study. It specifies standard portion sizes and caloric values for common food categories. It includes exemplar meal plans customized to varying daily energy needs, thus ensuring the standardization and reproducibility of the intervention protocol. [file JDR-2026-7230214-s001.pdf]

Calorie and Carbohydrate Restriction Dietary Guidance Manual

Name: ___________ Gender: □ Male □ Female

Date of Birth: _________ Age: ____ years Height: ____ cm Weight: ____ kg

Date of Diabetes Diagnosis: _________

Diagnosed Complications or Comorbidities (Multiple selections allowed): □ None □ Yes

□ Hypertension □ Hyperlipidemia

□ Cardiovascular Disease □ Cerebrovascular Disease

□ Diabetic Nephropathy □ Retinopathy

□ Diabetic Foot □ Peripheral Neuropathy

□ Lower Extremity Vascular Disease □ Others: _______________

Dietary Control

Total Daily Calories: _____ kcal/day. Convert the total daily calories into corresponding food types and quantities:

____ portions of Grains & Tubers; ____ portions of Vegetables & Fruits; ____ portions of Meat, Eggs & Legumes; ____ portions of Dairy; ____ portions of Fats & Oils.

**1** Exchange Portion - Grains & Tubers	25g Rice / 25g Flour / 25g Buckwheat Noodles / 35g Salted Bread / 35g Uncooked Noodles / 125g Potato / 125g Taro / 75g Sweet Potato / 200g Fresh Corn / 25g Mung Beans / 25g Soda Crackers / 150g Chinese Yam / 70g Fresh Soybeans / 150g Lotus Root
**1** Exchange Portion - Vegetables	500g Leafy Vegetables (e.g., Bok Choy, Celery, Crown Daisy, Spinach, Napa Cabbage) / 500g Winter Melon / 500g Mung Bean Sprouts / 500g Bitter Melon / 400g White Radish / 350g Luffa / 200g Carrot / 400g Eggplant / 600g Cucumber / 400g Cauliflower / 400g Bell Pepper
**1** Exchange Portion - Meat, Eggs & Legumes	15g Pork Strips / 50g Lean Pork or Beef / 50g Pork Ribs / 100g Rabbit Meat / 80g Yellow Croaker / 1 Egg (approx. 60g) / 100g Shrimp / 50g Dried Tofu / 150g Soft Tofu / 100g Firm Tofu 70g Chicken Breast / 100g Beltfish / 100g Crucian Carp
**1.5** Exchange Portions - Dairy	250ml Milk / 250ml Soy Milk (unsweetened) / 125g Plain Yogurt (unsweetened) / 25g Milk Powder
**1** Exchange Portion - Fats & Oils	10-15ml Cooking Oil (1 level tablespoon) / about 3 Walnut halves / about 10 Peanuts / 25g Sunflower Seeds / 40g Watermelon Seeds

Sample Diet Plan (1,400 kcal/day)

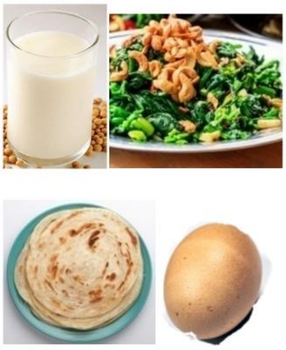	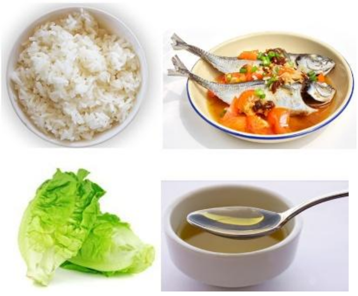	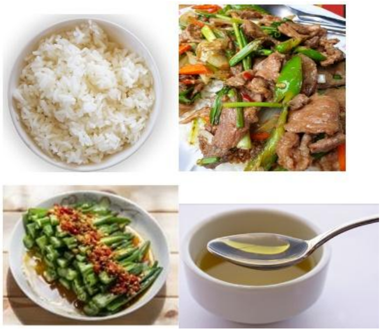
**Breakfast:**<br>• 250 ml pure milk<br>• 1 egg (70 g)<br>• 1 pancake (75 g flour)<br>• A small portion of cold mixed vegetables	**Lunch:**<br>• Cooked rice (75g raw weight)<br>• Steamed yellow croaker (80g raw weight)<br>• Stir-fried lettuce (250g raw weight)<br>• Cooking oil: 10–15 ml<br>• Salt: < 3g	**Dinner:**<br>• Cooked rice (75g raw weight)<br>• Sautéed pork slices with green pepper (pork: 50g raw weight)<br>• Okra (250g raw weight)<br>• Cooking oil: 10–15 ml<br>• Salt: <3g

Sample Diet Plan (1,600 kcal/day)

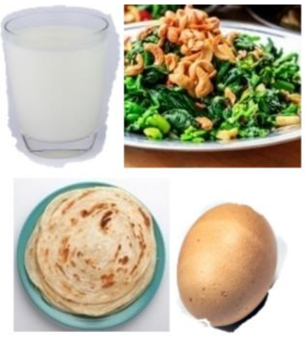	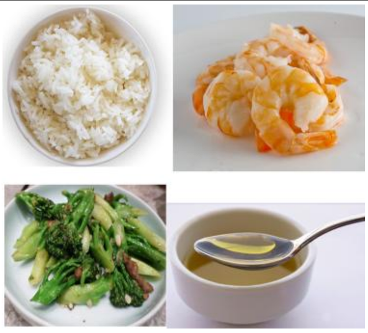	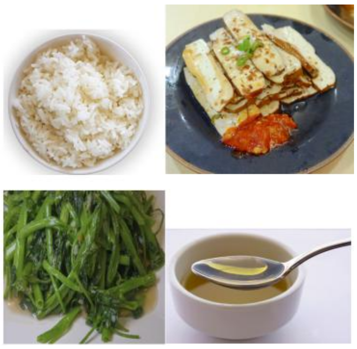
**Breakfast:**<br>• 250 ml soy milk<br>• 1 egg (70 g)<br>• 1 pancake (75 g flour)<br>• A small portion of cold mixed vegetables	**Lunch:**<br>• Cooked rice (100g raw weight)<br>• Prawns (100g raw weight)<br>• Stir-fried lettuce (250g raw weight)<br>• Cooking oil: 10-15ml<br>Salt: <3g	**Dinner:**<br>• Cooked rice (75g raw weight)<br>• Dried tofu (50g raw weight)<br>• Water spinach with garlic paste (250g raw weight)<br>• Cooking oil: 10-15ml<br>Salt: <3g

How to eat scientifically to help control blood sugar and ensure nutritional intake?

1. Control total daily calorie intake. Eat meals at regular times with fixed quantities. Diversify food types to obtain comprehensive nutrition.

2. Four major food categories are essential: grains & tubers, vegetables & fruits, meat, eggs & legumes, and fats & oils. Distribute them evenly across three main meals.

3. Combine whole grains and refined grains. Combine animal and plant-based foods. Eat until 70-80% full.

Eating Fruit Correctly and Appropriately:

- **Condition:** Blood sugar levels are stable and within the target range.
- **Time:** Between meals, for example, around 10:00 AM or 3:00 PM.
- **Types:** Choose fruits with low sugar content and control the portion size. Examples (to be consumed in two separate servings):

★ 200g Pomelo / 300g Strawberries / 200g Kiwi / 200g Apple / 200g Pear / 200g Plum / 300g Orange

- **Accounting:** Deduct the calories from the fruit (equivalent to 1 portion) from the total daily food energy intake. It is recommended to deduct this portion from the grains and tubers allowance.

Prevention and Management of Hypoglycemia:

1. Hypoglycemia is diagnosed when a diabetic patient's blood glucose level is **≤3.9 mmol/L**.
2. **Symptoms:** Tremors, palpitations, sweating; feeling of hunger; weakness and fatigue; in severe cases, convulsions and coma.
3. For individuals who are conscious: Follow the "Rule of 15"
   ① Stop all activity immediately. If a glucose meter is available, check blood sugar to confirm the level.
   ② Consume 15 grams of fast-acting carbohydrates, such as: 15 grams of glucose / 150 ml of fruit juice / 15 grams of hard candy / Two large sugar cubes / One tablespoon of honey.
   ③ Re-check blood sugar after 15 minutes.
   ④ If blood sugar is still ≤3.9 mmol/L, repeat the steps above until it returns to normal.
   ⑤ If hypoglycemia occurs frequently or for unknown reasons, consult your doctor.

To prevent hypoglycemia:

- Eat meals on a regular schedule with consistent portions.
- Take medication on time and as prescribed.
- Monitor blood sugar regularly.
- Engage in regular physical activity.
- Avoid excessive alcohol consumption and drinking on an empty stomach.
